# Feasibility and Acceptability of Internet-Based Interpersonal Psychotherapy for Stress, Anxiety, and Depression in Prenatal Women: Thematic Analysis

**DOI:** 10.2196/23879

**Published:** 2022-06-10

**Authors:** Katherine S Bright, Scott Stuart, Deborah A Mcneil, Lindsay Murray, Dawn E Kingston

**Affiliations:** 1 Faculty of Nursing University of Calgary Calgary, AB Canada; 2 Women's Mental Health Clinic Foothills Medical Centre Alberta Health Services Calgary, AB Canada; 3 Maternal Newborn Child & Youth Strategic Clinical Network Alberta Health Services Calgary, AB Canada

**Keywords:** internet-based, interpersonal psychotherapy, mental health, prenatal, anxiety, depression, stress, mobile phone

## Abstract

**Background:**

Prenatal mental health is a global health concern. Despite the far-reaching impact of prenatal mental health issues, many women do not receive the psychological care they require. Women in their childbearing years are frequent users of the internet and smartphone apps. Prenatal women are prime candidates for internet-based support for mental health care.

**Objective:**

This study aimed to examine the feasibility and acceptability of internet-based interpersonal psychotherapy (IPT) for prenatal women.

**Methods:**

Semistructured interviews were conducted with women who had received internet-based IPT modules with guided support as a component of a randomized controlled trial evaluating the scale-up implementation of a digital mental health platform (The Healthy Outcomes of Pregnancy and Postpartum Experiences digital platform) for pregnant women. Qualitative thematic analysis was used to explore and describe women’s experiences. Data were analyzed for emerging themes, which were identified and coded.

**Results:**

A total of 15 prenatal women were interviewed to examine their experiences and views on the feasibility and acceptability of internet-based IPT modules. Participants found the content informative and appreciated the ways in which the digital mental health platform made the IPT modules accessible to users. Participants voiced some differing requirements regarding the depth and the way information was presented and accessed on the digital mental health platform. The important areas for improvement that were identified were acknowledging greater depth and clarity of content, the need for sociability and relationships, and refinement of the digital mental health platform to a smartphone app.

**Conclusions:**

This study provides useful evidence regarding treatment format and content preferences, which may inform future development. It also provides research data on the feasibility and acceptability of web-based applications for prenatal mental health care.

**Trial Registration:**

ClinicalTrials.gov NCT01901796; https://clinicaltrials.gov/ct2/show/NCT01901796

## Introduction

### Background

Prenatal mental health is a global health issue, with 15% to 25% of pregnant women experiencing clinical levels of depression, anxiety, and stress [1-3]. Left untreated, prenatal psychological distress is associated with a range of negative consequences on obstetrical outcomes, maternal functioning, infant and child development, interpersonal conflict, parenting strain, and postpartum mood disorders [4-7]. Despite the far-reaching impact of prenatal mental health issues and well-established recommendations for routine mental health screening during the prenatal period, <20% of providers routinely screen [8]. Equally concerning is that only 1 in 7 prenatal women obtain the mental health intervention they require [8,9].

Interpersonal psychotherapy (IPT) is a highly effective treatment for depression and anxiety [10-12]. IPT improves symptoms of depression and anxiety in prenatal women [13-16]. Stuart and O’Hara [17] suggested that IPT is a mainline treatment for prenatal mental health as it focuses on addressing 4 interpersonal problems: role transitions, interpersonal disputes, grief, and interpersonal deficits. These 4 areas address the significant factors involved in the prediction and maintenance of depression and anxiety in pregnant women. The US Preventive Task Force has identified convincing evidence that IPT is effective in treating prenatal depression [18]. Although IPT is considered an effective treatment for depressive and anxiety disorders in the prenatal period, many women are uncertain about what is considered anticipated or expected mental health experiences during pregnancy, and as such, many women are reluctant to access mental health care [10,13,19-23]. Additional barriers to access to face-to-face IPT remain a challenge because of limited IPT-trained therapists, long wait times to access care, and the high cost of therapy sessions [24,25]. As a result, there is a need to make effective IPT interventions more readily available and accessible to pregnant women.

### Internet-Based Interventions for Prenatal Mental Health

Internet-based interventions are ideal treatment options for prenatal women to overcome major obstacles to accessing therapy, such as long wait times, busy schedules, stigmas to accessing care, and the financial burden associated with treatment [24]. In addition, internet-based interventions demonstrate preliminary effectiveness in the prevention [26,27] and treatment [28,29] of prenatal depression. Guided internet-based treatments have high levels of patient adherence and convincing reductions in mental health symptoms. They are readily available and cost-effective alternatives to face-to-face treatment [25,30]. Guided support, as a component of an internet-based treatment, has been reported in several systematic reviews to increase adherence and effectiveness for participants [31-33]. Guided support permits personal connections when needed and is a beneficial feature of internet-based interventions [34,35].

### The Healthy Outcomes of Pregnancy and Postpartum Experiences With the IPT Program

We developed an internet-based prenatal mental health intervention for a low-intensity, guided support IPT program for stress, anxiety, and depression. The IPT program comprises 6 modules adapted from the IPT clinician guide developed by Stuart and Robertson [36] and tailored to the specific needs of prenatal women. These IPT modules were delivered through a digital mental health platform (The Healthy Outcomes of Pregnancy and Postpartum Experiences [HOPE] digital platform). Given the model of IPT developed by Stuart and Robertson [36] as benefiting problems involving role transitions, loss, and interpersonal conflict, 6 modules were built around these 3 areas [20]. Role transitions during the prenatal and postpartum periods are primarily related to developing new skills and accommodating changing responsibilities while maintaining relationships. This problem area explores the multiple roles women juggle and the increased relationship demands because of these roles [17,36]. Women are encouraged to combine new roles rather than give up old roles, express emotions attached to each of the roles and their impact on self-esteem, and explore ambivalent feelings for each role. Ultimately, the intention of exploring role transitions is to assist women in developing a more balanced understanding of each role, modifying expectations, and assisting them in restructuring priorities [17,36].

The focus area of loss explores grief reactions that coincide with pregnancy and the arrival of a newborn [36]. The therapeutic intent of exploring loss is to assist with the mourning process and help women cultivate new and current relationships that can be substituted for relationships that were lost [17,36]. The focus area of interpersonal conflict is one of the most significant possible stressors during pregnancy and into the postpartum period. This often occurs in individuals and their spouses [17,36].

This internet-based prenatal mental health intervention comprises six 30-minute, web-based IPT modules delivered over 6 to 8 weeks. The topics of the modules are (1) identifying the important relationships in women’s lives, (2) understanding and improving communication patterns, (3) navigating interpersonal disputes, (4) adapting to role transitions, (5) working through grief and loss, and (6) maintaining IPT strategies and carrying these skills forward in their lives after the study ends. These topics were tailored to pregnant women. Participants were asked to complete specific assignments on the web for each module, such as self-awareness homework and exploring relationships with those close to them. The goals of the intervention included symptom relief, improving interpersonal functioning and relationships, changing expectations about interpersonal relationships, and improving social support networks. Participants were guided to recognize and disengage from unhelpful communication patterns and foster strategies for developing and nurturing social support in navigating challenging times, such as role transitions, conflict with their partner or extended family members, and grief or loss.

Within this program, women were encouraged to assess their expectations for roles that they, their partners, parents, in-laws, and other children have during the prenatal period. Within these expectations, women explore the changes and consistency of roles before, during, and after pregnancy into the postpartum period. A significant aspect of the focus area of interpersonal conflict includes the identification of disputes and the development of problem-solving approaches that women can put into action. In addition, this IPT module contained the following components: development of a support system, effective communication strategies, and skills for managing conflict in relationships [36]. Homework exercises involved assessing attachment style; communication style; breaking down a distressing conversation or interaction; understanding one’s relationships through relationship circles; visualizing, describing, and resolving conflict through a disagreement or dispute graph; and understanding role transitions through a life events timeline. Women were assessed for the areas of life challenges that caused emotional distress. This provided a targeted direction regarding modules that best suited their needs (eg, loss, transition, and interpersonal).

The IPT internet-based prenatal mental health intervention, comprising 6 IPT modules, was a component of a randomized controlled trial that evaluated the scale-up of a digital mental health platform for pregnant women—the HOPE digital mental health platform.

This study aimed to investigate women’s views of the feasibility and acceptability of IPT internet-based prenatal mental health modules delivered through a digital mental health platform with low-intensity, guided support.

## Methods

### Overview

Semistructured interviews were conducted in a flexible and responsive manner as a method of data collection. Qualitative content analysis and thematic analysis were used as methods of data analysis. These qualitative interviews sought to understand women’s individual experiences of interacting with the digital mental health platform and address questions regarding the feasibility and acceptability of the internet-based IPT intervention.

### Design

A qualitative design was used to assess women’s individual experiences regarding the feasibility and acceptability of internet-based IPT interventions. This study adopted a pragmatic approach.

### Recruitment

A total of 20 participants in the intervention group were contacted by email to assess their interest in participating in an interview and then followed up by email to set up a suitable time for the telephone interview. Of the 20 participants, 15 (75%) women in the intervention group agreed to be interviewed by a member of the research team (KSB). Before the interviews, information on computer-generated program use was collected. Participants who completed more than half of the modules were considered *high users*, and those who completed less than half of the modules were considered *low users*. We also identified participants’ levels of engagement and explored the barriers to and facilitators of engagement.

### Data Collection

Semistructured interviews, lasting 45 to 75 minutes, were conducted in a flexible and responsive manner as a method of data collection. These interview questions were piloted with 3 prenatal women who were not in the study to ensure that the questions were clear and concise. The interviews addressed key areas based on guidelines for assessing internet intervention research [37]: assessment and navigation of the intervention, acceptability and perceived usefulness of various components of the intervention, and recommendations for improvement [38,39]. The interview questions were used as a guide; however, the interviews were conducted with the flexibility to allow participants to freely discuss topics. Recruitment continued during data analysis to achieve saturation of the themes. All interviews were conducted over the phone, audio recorded, and conducted by the first author (KSB; she or her), an experienced qualitative research interviewer, as a part of her doctoral dissertation and transcribed verbatim. KSB is a clinician in an outpatient reproductive psychiatric clinic for the past 10 years. Field notes were made following each interview. Participants received a CAD $5 (US $4) coffee card as compensation for their time.

### Analysis

Transcripts were coded and analyzed using thematic analysis techniques to identify categories and themes [40,41]. The approach used for this thematic analysis was guided by the method described by Braun and Clarke [40]. The semistructured interview guide provided a focused direction for the interviews. The themes that concurrently emerged from these interviews were relevant to the aim of our study and our research questions. A coding framework was developed in response to the themes that emerged during each interview. This coding framework was reviewed and refined as it was applied to data. Patterns within and across themes were explored using an analysis process. The main coding categories paralleled the questions asked during the interviews. Categories were also reflective of emerging trends in the data, which became apparent from the frequency of certain categories and repetition of points. Agreement on the categories and concepts was sought between members of the research team to ensure reliability. The interviews and coding framework were examined until no new information emerged from the data. KB, who has a background in qualitative interviewing, conducted the interviews and was the lead in the data analysis. LM and DEK, who have backgrounds in psychology, mental health, and the prenatal population, each read 5 interview transcripts. The coding framework was discussed throughout its development, with regular meetings between the 3 researchers to ensure that the concepts were appropriately identified and described.

### Ethics Approval

This study was registered with ClinicalTrials.gov (NCT01901796) and was approved by the University of Calgary Research Ethics Board (REB16-0061 U of Calgary).

## Results

### Overview

Of the 20 approached women, 15 (75%) agreed to participate in the interviews. Of the 15 participants, 10 (67%) were considered high users of the IPT modules, and the remaining 5 (37%) were classified as low users. The women ranged in age from 28 to 38 years. All women were partnered, and 67% (10/15) of women had ≥1 child.

The themes identified were feasibility and acceptability. Women reported the feasibility of the digital mental health platform (the HOPE platform) and IPT modules in 3 subthemes: treatment feasibility, flexible access, and impact of mental health status on platform engagement. Treatment feasibility refers to the ease with which the platform was integrated into women’s lives. Acceptability also had 3 subthemes: clarity and depth of content, lack of relationships with the internet-based IPT, and suggestions for improvement.

### Feasibility

Overall, women indicated that the delivery of internet-based IPT modules through the HOPE digital mental health platform was a way for them to self-manage their mental health during their pregnancy. Participants reported that they were pleased to find a mental health resource specifically designed for pregnant women. IPT modules and the platform were easily accessible at times and places that were convenient to the participants.

#### Treatment Feasibility

Overall, the women reported that the features of the internet-based IPT modules and platform appeared at the right time in their lives to be easily integrated into their lives and practices. Women reported that they wanted to participate in this internet-based IPT study as it was an opportunity to access support and information. They also reported an interest in accessing resources to assist with the transition through pregnancy and into the postpartum period:

I was looking for an independent way to learn more about what I was going through in pregnancy.

P1

I have mental health issues so I knew that it would be a good support for me and I just want to find out more information and more resources to just help me with this transition time, with pregnancy. It was, just kind of encouraging to see, the support there.

P2

I do know myself pretty well and know that I have a tendency to be more of an anxious person so I thought this might be a good opportunity to be proactive with my mental health.

P6

I think the biggest thing was that it was a resource specifically targeted to exactly where I was in my life, just in the middle of my pregnancy, kind of near the end, things that you are thinking about at that time mental health wise. It was just clearly targeted for where I was at.

P8

I joined this study because I have a history of postpartum depression with my first daughter so I figured I could use all the support I could find with my next pregnancy...I thought that it is good, we need more research into that.

P11

I had experienced depression one time before and I was looking for a resource that would help prevent postpartum depression. This program seemed hopeful. It targeted the time in pregnancy and that was exactly where I was at.

P14

#### Flexible Access

Participants enjoyed that the internet-based IPT modules could be accessed from home or at a location and time that suited them. This was reported as important as most women (14/15, 93%) were either working outside the home and inside the home with one or more children:

I’m actually able to do more exercises and modules after work.

P5

It was the perfect amount of time to complete them. Like you could logon on your lunchbreak and go through a module. That is what I did.

P6

I completed the modules and surveys on my phone. It was easy to do anywhere when I had the time...If you want to make the time for it you will do it.

P11

Web-app made it easy to use and access from anywhere.

P12

I quite like being in the comfort of my own home, logging on when I can if I can...I do like that they (the modules) were short and sweet...I found that shortness of the modules was really important for me to start and finish and get something out of it rather than just get part way through it and have to stop.

P13

#### Impact of Mental Health Status on Platform Engagement

A few participants reported that their mental health status at the moment predicted their engagement on the platform and their motivation to work through the modules. When they were doing well mentally, they reported that they were able to work through the internet-based IPT modules. When they felt they were not doing well, they did not access the platform modules:

When I am good, I’m good, right? When I’m bad, it’s the complete opposite. That I totally shut down and that’s when you need something the most.

P4

I have so much stuff going on right now, I am 6 months pregnant, working, and feeling really anxious. To tell you the truth I kind of forgot about the Web-App and modules. It has been a couple of months and I think it would have been helpful to have a reminder every day to cue me back to it.

P5

It was hard to find the time to really pay attention and I think I was also a little bit too deep into my mental health issues at that time to really be able to appreciate the app and modules for what they were.

P5

### Acceptability

#### Overview

Participants reported that they found the digital mental health platform (the HOPE platform) and IPT modules user-friendly and helpful for increasing their skills to solve real-life concerns. The participants greatly appreciated the guided support or coaching aspect of the digital platform. Most of the mental health platform users reported that they would have preferred the digital platform to be a smartphone app—an app that can be accessed on their phone rather than having to access the IPT modules through a browser over the internet. Many participants reported that the modules contained information that was relevant to them, and they wished that the modules had been developed in greater depth. Although participants enjoyed the HOPE digital mental health platform and IPT modules, they missed the person-to-person connections that occur with individual and group therapy.

#### Usefulness

Overall, participants reported that the internet-based IPT modules were acceptable, displayed well on their smartphones or computers, and were user-friendly and modern:

I actually appreciated that they were very easy to interact with. I didn’t feel like I was lost looking for buttons or cues or having to read things again.

P1

I thought the interface was pretty clear and pretty easy to interact with. I could record my answers quite easily.

P2

The interface of the app was easy to use.

P9

Women reported that the content in the modules was useful, relevant for their current needs, increased their self-awareness, and helped them solve real-life problems:

It was very useful actually. I’ve never sat down and really understood how my tendencies would impact the way that I was interacting with my husband specifically.

P1

I think one of the most import parts of the modules was communication skills and being assertive...I liked how it gave you an example of how it could help in real life...I am keeping in mind some of the communication pieces as we are navigating some of the postpartum issues.

P6

I liked how the modules really made you think about what is going on for you. They are definitely teaching you things that make you take a different look at what is going on in your life.

P7

I think that the biggest benefit of the modules was just a different way of thinking of situations differently or providing coping tools to address the here and now...I definitely found them helpful.

P9

I am currently having some issues in a friendship, and I think that the relationship module is going to be really helpful in resolving this.

P11

I really liked that I could have a project to work on, something a little more concrete to work though because having something concrete is helpful for me to learn.

P12

Many participants reported the benefits of the guided support or coach component of the digital mental health platform:

I liked the coach. That was probably my favorite part. It’s just having that someone that just checked in...it’s just that extra check and like I said, you know, if you’re not comfortable going to a friend.

P4

I think that the biggest benefit to me was when I put down the need for a call, somebody actually did call me...It was nice to just have somebody on the other end of the line who was like we saw that you wanted to be called by a coach...It was really nice to have someone reach out and see how I was doing and to see what resources I needed.

P9

I talked to the coach for about 45 minutes, and it was really helpful. She did a fantastic job of talking with me about what was going on for me.

P12

I was contacted by the coach and that was really good. I didn’t need any resources or anything, but it was nice to have her check in on me. I found that really helpful.

P14

I had a call from the coach, and I found that more helpful...Just having someone to talk who already knows I am pregnant and already knows that I am in a very vulnerable state during pregnancy, and they knew the app better than I did and they could tell me where to go or what local resources are available.

P15

All participants stated that they would prefer the IPT modules to be delivered through a smartphone app rather than the web application:

The one thing would be more accessible would be if it was just an app that you can download. To use on the go.

P2

I think a mobile app would really help. Like I don’t really even check my email anymore. I think a true app would really help with that.

P6

I would have preferred to see this in an app form instead of a Web-App.

P8

It was a little disappointing that is wasn’t just an app that I could find on my phone and that I had to log on through a web browser.

P10

I thought it was an App, but it was a Web-App. That was a bit misleading. Either change the name to Web-App or make it an App.

P11

#### Clarity and Depth of Content

Of the 15 participants, 12 (80%) reported that the content of the IPT modules required further refinement. These participants reported that all topics were relevant; however, they wanted to go further, recommending a greater depth of material:

It was nothing new, I kind of knew, like, information about attachment style like its nothing I haven’t heard before. So, it was good there were a couple of examples of case studies, Yeah, like I said but it’s very, kind of, basic.

P2

It wasn’t necessarily new information.

P4

I think for me sometimes it was a bit generic or really surface level and I understand that in these modules you can’t really get into the nitty gritty but maybe it is because of my background that I was like I kind of knew that.

P6

I felt like the modules were really short, so you kind of got into a topic, and some of them were really useful and helpful, but they seemed really brief. Like they could have gone into more detail.

P8

I think if you had a list of additional resources in the modules for more information if you need it or if you really wanted to go further.

P10

The introductory ones seemed ok but pretty basic. But the one that I have stopped at, the one that has an exercise, and I am hoping that the future ones are more like that because it seems like they will actually be more useful and have new tools for me.

P11

I felt there the information was good but there was nothing like to go from here. Like say with the attachment style one, I really liked learning about attachment styles, but it didn’t really give me, and I wanted more information like this is how your approach your partner and this is how your partner approaches you. Like I wanted to know what I was and then I wanted to have tools and I felt like I didn’t that I was given the toolbox but not the tools.

P12

A lot of the information was not new to me but the reminder of it is still really important.

P13

Many participants reported that the modules required further refinement of the language used throughout and clarity of instructions for exercises or homework:

The verbiage in a couple of the modules do have the inclination, to be that way is bad and to be this way is the better option...to do an honest assessment of where they are at, its understanding that your communication style and your attachment style is never bad it’s just where you are right now.

P3

I think that is the area where I struggled most. Sometimes I wasn’t even clear what I was supposed to do or what the homework was.

P6

That’s the part that I am stuck on now. I remember not being able to fully understand it. And I think that is a big reason why I wasn’t able understand what I could take from what is going on in my life right now and to be able to implement it to complete the exercise. I needed a better understanding of what the homework was asking because I wasn’t understanding what it was asking.

P7

I think that if the app could have it so you could do a lot of the exercises in real time so actually inputting your thoughts and your data into app and it could organize it for you. I think that would make it a lot more attractive to the user.

P9

I think clearly outlining what is available on this Web-App from the beginning would help like the surveys, the modules, the mood tracker, and things like that. And giving people an idea of what is going to happen when so that it is not forgotten or confusing when things come up.

P10

I have competed over half of the modules, but I am stuck on one because it asks you to do a homework assignment outside of the app and I am stuck. I don’t want to blow past in and not do it. I need to sit down and make the time to do it. It just hasn’t happened yet.

P11

I think that the modules could focus on how exactly to ask for help from others.

P13

#### Personal Connection

Participants reported that although they enjoyed the ease of the internet-based IPT modules and screening, they missed the opportunity to connect with other individuals:

I remember just thinking I needed more and that is where the personal sessions would help a lot more.

P11

I am a big advocate for people helping people. I appreciate the ease of online work but also miss the face-to-face interaction you get with a therapist...Maybe online you need to offer both for people to have the option to do it on their own or to login into a closed group if they want, appealing to different personalities.

P13

It is hard for me to properly put down a number indicating how I feel because it is based on the day, so I just like it was very generic and I am not really sure if it got, understood where I was coming from. It was more helpful for me once I got a hold of someone to talk to. It was the personal connection that was important to me...Technology is great, but it can only do so much.

P15

#### Suggestions for Improvement

Suggestions for improvement for many women included incorporating different learning styles:

I find that I learn a lot by watching things too. Like there is so much on YouTube and TedTalks to find helpful, like Brené Brown or something cool like that would be something for interpersonal relationships and vulnerability...It is about catering to different learning styles.

P2

I am a very visual learner so having videos instead of a lot of reading would have been more helpful for me.

P7

I used multiple apps during my pregnancy it would have been nice to have one that tracks where I am in my pregnancy and mental health to see if they could be linked or incorporated into one app would be something that I would be interested in.

P8

Having the modules as an audio, like something I could listen to while driving, might be helpful.

P15

In addition, the participants suggested embedding mental health into a pregnancy app to include mental health as a part of overall health:

I think it would be helpful to integrate this app into a pregnancy app, like all of us had the bump app, and it kind of goes through some of the physical changes in pregnancy and I think people are kind of curious about that the baby is doing and what their bodies are doing, so that might be nice to tie together. That is more holistic too. So, showing people that mental health is just health.

P6

In addition, there was a suggestion to extend the IPT work into the postpartum period with modules aimed at the challenges of being a new parent:

I think it would be helpful to have additional modules to work through in the postpartum period. Like navigating yourself, your new baby, and your relationship with your partner in the postpartum period.

P12

Overall, the participants found the HOPE digital mental health platform and IPT modules helpful in managing their mental health concerns during pregnancy. They also had many suggestions to further improve the HOPE digital mental health platform and IPT modules. These women suggested that the platform and modules could attend to a variety of learning styles, including the use of videos and audio components. In addition, the participants wanted this mental health component to be integrated into existing pregnancy apps as part of comprehensive prenatal and postpartum care.

## Discussion

### Principal Findings

This study aimed to assess the feasibility and acceptability of a pilot study of internet-based IPT modules. Regarding the feasibility of the study, participants reported that the features of the internet-based IPT modules delivered via the digital mental health platform (the HOPE digital platform) were easily integrated into their lives. Of the 15 participants, 10 (66%) were described as high users of the modules, completing >4 of the 8 modules. The internet-based delivery of the IPT modules permitted flexible and independent access. Participants appreciated being able to seek support and resources to self-manage the emotional or mental health challenges arising during their pregnancies.

Participants reported that the format of the digital mental health platform was acceptable and user-friendly. They also reported that the digital mental health platform could be improved by delivering the format through a smartphone app. Among the women who accessed the internet-based IPT, the treatment was considered generally useful and helpful in examining communication and interactions with others. Many women reported that the modules were helpful in solving concrete problems. Although described as useful, there were elements of the modules that the participants felt could be improved. Some considered the information to be too basic and required further refinement. They also sought improvement in the depth and breadth of content and clarity of instructions for exercises and homework. In addition, the participants suggested that they would benefit from additional directions on what they needed to do when not managing well.

Participants reported that they appreciated the guided support or coach aspect of the internet-based IPT modules and digital mental health platform. Whether the coach was triggered in the digital mental health platform to contact women (based on responses to survey questionnaires) or the women requested a coach callback, the guided support or coach gave women the feeling that they were *being checked in on*. They valued having the coach see how they were doing. Participants reported that this was one of the most important aspects of the IPT modules and digital mental health platform.

### Comparison With Prior Work

This study found that an internet-based mental health intervention was feasible, with participants citing the ease and flexibility of access as beneficial. These results are consistent with previous studies [42-45]. As mentioned by the participants in this study, a smartphone app is preferred over the delivery of interventions with a web application. Previous studies have also reported an increase in treatment accessibility and participation in mental health interventions when delivered via smartphone apps [46]. Furthermore, internet-based and smartphone apps provide convenient and potentially anonymous access to treatment. Smartphone apps can also overcome conventional barriers to seeking help, including lack of time, stigma, childcare issues, and embarrassment [47-49].

The efficacy of internet-based interventions is well-established for various mental health disorders, including depression, anxiety, agoraphobia, panic disorder, and stress [25,50,51]. Acceptability of internet-based interventions has been reported across different populations, including students [52], perinatal women [53], and older adults [54].

This study adds to the evidence that pregnant women seek resources and support to self-manage mild to moderate mental health concerns [55]. Consistent with previous studies, the preference of women to address mental health problems on their own highlights the need and opportunity to offer alternatives to traditional face-to-face, therapist-led interventions [56-58]. Providing prenatal women with effective resources and support to self-manage their mild to moderate mental health concerns, including internet-based therapy, may be an effective approach for pregnant women with mild to moderate mental health concerns.

This study was unique in that mental health symptoms may impact engagement in the digital mental health platform. Participants in this study reported that they were less inclined to access the web application platform when they were doing very well and when they were struggling with significant psychological distress. This finding seems to contradict the findings that mental health symptoms had no relationship with a preference for and engagement in web-based mental health programs [59,60]. These studies indicate that neither high nor low symptoms of depression and anxiety caused individuals to be more or less likely to engage in web-based mental health interventions.

Guided support is a beneficial feature of internet-based interventions; this preference for person-to-person support is consistent with previous work [34,35]. However, evidence regarding the nature of guided support is lacking. The use of face-to-face or person-to-person mental health programs is slightly preferred for those experiencing emotional and personal problems [61-64]. Predictors for the preference of person-to-person mental health interventions included currently experiencing emotional or personal problems. The use of coach-based guided support along with mental health apps for smartphones has been found to improve mental and emotional health by decreasing participants’ depression, anxiety, and general distress [46,65-67]. Guided support or coaching with internet-based mental health interventions has been reported to be an effective, cost-efficient, and acceptable alternative to face-to-face therapy [33]. Systematic reviews exploring the internet-based mental health interventions found that offering some form of support or guidance during the web-based treatment increased the effectiveness and was associated with higher levels of program completion [34,68]. Findings from this study will help move this area of study forward by identifying the importance of coach-based guided support within a pregnant population. This study may lead to an increase in the refinement of smartphone delivery of IPT in pregnant women.

### Limitations

The findings of the usability, feasibility, and acceptability of internet-based IPTs are limited to the experiences and perspectives of a small sample. None of the women who withdrew from the study before completing the first module reported reasons for dropping out of the program.

The research team took several actions to minimize the likelihood of bias during the analysis phase. The research team had regular discussions about the coding framework with fellow team members who each reviewed 5 interviews to ensure that the concepts were being appropriately identified and described.

Participants who agreed to be interviewed were offered a small monetary compensation for their time, which may have affected their responses.

Another limitation may be the generalizability of these findings because of the smaller prenatal sample size.

### Clinical Implications

This study provides insights into the acceptability of this internet-based IPT program for prenatal women. This information could be used to further refine the development of targeted IPT mental health support for women during their pregnancy and into the postpartum period. Smartphone app development is required for the delivery of IPT programs. This will support women during their pregnancy and into the postpartum period. Future smartphone IPT programs aimed at prenatal women would also benefit from guided or coach support components.

### Conclusions

This study demonstrated that there is a significant interest in prenatal mental health internet-based IPT treatment. The current format of this internet-based IPT needs refinement. This study revealed the strengths and weaknesses of the content and format of this internet-based IPT. In addition, the study participants highlighted potential areas of improvement. The IPT modules for women during the prenatal period would benefit from the further refinement of the content and integration of the revised modules into a randomized controlled trial to explore the efficacy of the internet-based IPT modules on perinatal mental health concerns.

## Abbreviations

HOPE: Healthy Outcomes of Pregnancy and Postpartum Experiences
IPT: interpersonal psychotherapy

## References

[1] Goodman JH, Tyer-Viola L (2010). Detection, treatment, and referral of perinatal depression and anxiety by obstetrical providers. J Womens Health (Larchmt).

[2] Khan L Falling through the gaps: perinatal mental health and general practice. Centre for Mental Health.

[3] O'Hara MW, Wisner KL (2014). Perinatal mental illness: definition, description and aetiology. Best Pract Res Clin Obstet Gynaecol.

[4] Tomfohr L, Buliga E, Letourneau N, Campbell T, Giesbrecht GF (2015). Trajectories of sleep quality and associations with mood during the perinatal period. Sleep.

[5] Kozyrskyj AL, Letourneau NJ, Kang LJ, Salmani M (2017). Associations between postpartum depressive symptoms and childhood asthma diminish with child age. Clin Exp Allergy.

[6] Lebel C, Walton M, Letourneau N, Giesbrecht GF, Kaplan BJ, Dewey D (2016). Prepartum and postpartum maternal depressive symptoms are related to children's brain structure in preschool. Biol Psychiatry.

[7] Letourneau NL, Dennis CL, Benzies K, Duffett-Leger L, Stewart M, Tryphonopoulos PD, Este D, Watson W (2012). Postpartum depression is a family affair: addressing the impact on mothers, fathers, and children. Issues Ment Health Nurs.

[8] Kim JJ, La Porte LM, Corcoran M, Magasi S, Batza J, Silver RK (2010). Barriers to mental health treatment among obstetric patients at risk for depression. Am J Obstet Gynecol.

[9] Bowen A, Bowen R, Butt P, Rahman K, Muhajarine N (2012). Patterns of depression and treatment in pregnant and postpartum women. Can J Psychiatry.

[10] Cuijpers P, Geraedts AS, van Oppen P, Andersson G, Markowitz JC, van Straten A (2011). Interpersonal psychotherapy for depression: a meta-analysis. Am J Psychiatry.

[11] Donker T, Bennett K, Bennett A, Mackinnon A, van Straten A, Cuijpers P, Christensen H, Griffiths KM (2013). Internet-delivered interpersonal psychotherapy versus internet-delivered cognitive behavioral therapy for adults with depressive symptoms: randomized controlled noninferiority trial. J Med Internet Res.

[12] Stangier U, Schramm E, Heidenreich T, Berger M, Clark DM (2011). Cognitive therapy vs interpersonal psychotherapy in social anxiety disorder: a randomized controlled trial. Arch Gen Psychiatry.

[13] Spinelli MG (1997). Interpersonal psychotherapy for depressed antepartum women: a pilot study. Am J Psychiatry.

[14] Spinelli MG, Endicott J (2003). Controlled clinical trial of interpersonal psychotherapy versus parenting education program for depressed pregnant women. Am J Psychiatry.

[15] Brandon AR, Ceccotti N, Hynan LS, Shivakumar G, Johnson N, Jarrett RB (2012). Proof of concept: partner-assisted interpersonal psychotherapy for perinatal depression. Arch Womens Ment Health.

[16] Sockol LE, Epperson CN, Barber JP (2011). A meta-analysis of treatments for perinatal depression. Clin Psychol Rev.

[17] Stuart S (2012). Interpersonal psychotherapy for postpartum depression. Clin Psychol Psychother.

[18] Curry SJ, Krist AH, Owens DK, Barry MJ, Caughey AB, Davidson KW, Doubeni CA, Epling JW, Grossman DC, Kemper AR, Kubik M, Landefeld CS, Mangione CM, Silverstein M, Simon MA, Tseng CW, Wong JB, US Preventive Services Task Force (2019). Interventions to prevent perinatal depression: US Preventive Services Task Force Recommendation statement. JAMA.

[19] Cuijpers P, van Straten A, Andersson G, van Oppen P (2010). Psychotherapy for depression in adults: a meta-analysis of comparative outcome studies. Focus.

[20] Donker T, Batterham P, Warmerdam L, Bennett K, Bennett A, Cuijpers P, Griffiths K, Christensen H (2013). Predictors and moderators of response to internet-delivered Interpersonal Psychotherapy and Cognitive Behavior Therapy for depression. J Affect Disord.

[21] Markowitz JC, Lipsitz J, Milrod BL (2014). Critical review of outcome research on interpersonal psychotherapy for anxiety disorders. Depress Anxiety.

[22] Stuart S, Clark E (2008). [The treatment of postpartum depression with interpersonal psychotherapy and interpersonal counseling]. Sante Ment Que.

[23] Gennaro S, OʼConnor C, McKay E, Gibeau A, Aviles M, Hoying J, Melnyk BM (2020). Perinatal anxiety and depression in minority women. MCN Am J Matern Child Nurs.

[24] Kingston D, Tough S (2014). Prenatal and postnatal maternal mental health and school-age child development: a systematic review. Matern Child Health J.

[25] Andrews G, Cuijpers P, Craske MG, McEvoy P, Titov N (2010). Computer therapy for the anxiety and depressive disorders is effective, acceptable and practical health care: a meta-analysis. PLoS One.

[26] Barrera AZ, Wickham RE, Muñoz RF (2015). Online prevention of postpartum depression for Spanish- and English-speaking pregnant women: a pilot randomized controlled trial. Internet Interv.

[27] Haga SM, Drozd F, Brendryen H, Slinning K (2013). Mamma mia: a feasibility study of a web-based intervention to reduce the risk of postpartum depression and enhance subjective well-being. JMIR Res Protoc.

[28] Danaher BG, Milgrom J, Seeley JR, Stuart S, Schembri C, Tyler MS, Ericksen J, Lester W, Gemmill AW, Lewinsohn P (2012). Web-based intervention for postpartum depression: formative research and design of the MomMoodBooster program. JMIR Res Protoc.

[29] O'Mahen HA, Richards DA, Woodford J, Wilkinson E, McGinley J, Taylor RS, Warren FC (2013). Netmums: a phase II randomized controlled trial of a guided Internet behavioural activation treatment for postpartum depression. Psychol Med.

[30] Andrews G, Titov N (2010). Is internet treatment for depressive and anxiety disorders ready for prime time?. Med J Aust.

[31] Spek V, Cuijpers P, Nyklícek I, Riper H, Keyzer J, Pop V (2007). Internet-based cognitive behaviour therapy for symptoms of depression and anxiety: a meta-analysis. Psychol Med.

[32] Titov N (2011). Internet-delivered psychotherapy for depression in adults. Curr Opin Psychiatry.

[33] Andersson G, Cuijpers P, Carlbring P, Riper H, Hedman E (2014). Guided internet-based vs. face-to-face cognitive behavior therapy for psychiatric and somatic disorders: a systematic review and meta-analysis. World Psychiatry.

[34] Richards D, Richardson T (2012). Computer-based psychological treatments for depression: a systematic review and meta-analysis. Clin Psychol Rev.

[35] Johansson R, Andersson G (2012). Internet-based psychological treatments for depression. Expert Rev Neurother.

[36] Stuart S, Robertson M, Robertson M (2012). Interpersonal Psychotherapy 2E A Clinician's Guide.

[37] The Hope Project. Dr. Dawn Kingston.

[38] Proudfoot J, Klein B, Barak A, Carlbring P, Cuijpers P, Lange A, Ritterband L, Andersson G (2011). Establishing guidelines for executing and reporting internet intervention research. Cogn Behav Ther.

[39] Rozental A, Andersson G, Boettcher J, Ebert DD, Cuijpers P, Knaevelsrud C, Ljótsson B, Kaldo V, Titov N, Carlbring P (2014). Consensus statement on defining and measuring negative effects of internet interventions. Internet Interventions.

[40] Braun V, Clarke V (2006). Using thematic analysis in psychology. Qual Res Psychol.

[41] Nowell LS, Norris JM, White DE, Moules NJ (2017). Thematic analysis: striving to meet the trustworthiness criteria. Int J Qual Methods.

[42] Chan JK, Farrer LM, Gulliver A, Bennett K, Griffiths KM (2016). University students' views on the perceived benefits and drawbacks of seeking help for mental health problems on the internet: a qualitative study. JMIR Hum Factors.

[43] Carter J, Sandall J, Shennan AH, Tribe RM (2019). Mobile phone apps for clinical decision support in pregnancy: a scoping review. BMC Med Inform Decis Mak.

[44] Battle JD, Farrow L, Tibaijuka J, Mitchell M (2015). mHealth for Safer Deliveries: a mixed methods evaluation of the effect of an integrated mobile health intervention on maternal care utilization. Healthc (Amst).

[45] Marko KI, Krapf JM, Meltzer AC, Oh J, Ganju N, Martinez AG, Sheth SG, Gaba ND (2016). Testing the feasibility of remote patient monitoring in prenatal care using a mobile app and connected devices: a prospective observational trial. JMIR Res Protoc.

[46] Donker T, Petrie K, Proudfoot J, Clarke J, Birch MR, Christensen H (2013). Smartphones for smarter delivery of mental health programs: a systematic review. J Med Internet Res.

[47] O'Mahen HA, Flynn HA (2008). Preferences and perceived barriers to treatment for depression during the perinatal period. J Womens Health (Larchmt).

[48] Woolhouse H, Brown S, Krastev A, Perlen S, Gunn J (2009). Seeking help for anxiety and depression after childbirth: results of the Maternal Health Study. Arch Womens Ment Health.

[49] Dennis C, Chung-Lee L (2006). Postpartum depression help-seeking barriers and maternal treatment preferences: a qualitative systematic review. Birth.

[50] Barak A, Hen L, Boniel-Nissim M, Shapira N (2008). A comprehensive review and a meta-analysis of the effectiveness of internet-based psychotherapeutic interventions. J Technol Human Services.

[51] Barak A, Klein B, Proudfoot JG (2009). Defining internet-supported therapeutic interventions. Ann Behav Med.

[52] Farrer L, Christensen H, Griffiths KM, Mackinnon A (2012). Web-based cognitive behavior therapy for depression with and without telephone tracking in a national helpline: secondary outcomes from a randomized controlled trial. J Med Internet Res.

[53] Fonseca A, Gorayeb R, Canavarro MC (2016). Women's use of online resources and acceptance of e-mental health tools during the perinatal period. Int J Med Inform.

[54] Preschl B, Wagner B, Forstmeier S, Maercker A (2011). E-health interventions for depression, anxiety disorders, dementia, and other disorders in old age: a review. J Cyber Ther Rehabil.

[55] Kingston DE, Biringer A, McDonald SW, Heaman MI, Lasiuk GC, Hegadoren KM, McDonald SD, van Zanten SV, Sword W, Kingston JJ, Jarema KM, Vermeyden L, Austin M (2015). Preferences for mental health screening among pregnant women: a cross-sectional study. Am J Prev Med.

[56] Kingston D, Austin M, Heaman M, McDonald S, Lasiuk G, Sword W, Giallo R, Hegadoren K, Vermeyden L, van Zanten SV, Kingston J, Jarema K, Biringer A (2015). Barriers and facilitators of mental health screening in pregnancy. J Affect Disord.

[57] Reay R, Matthey S, Ellwood D, Scott M (2011). Long-term outcomes of participants in a perinatal depression early detection program. J Affect Disord.

[58] Goodman JW (2009). Women's attitudes, preferences, and perceived barriers to treatment for perinatal depression. Birth.

[59] Batterham PJ, Calear AL (2017). Preferences for internet-based mental health interventions in an adult online sample: findings from an online community survey. JMIR Ment Health.

[60] Wallin EE, Mattsson S, Olsson EM (2016). The preference for internet-based psychological interventions by individuals without past or current use of mental health treatment delivered online: a survey study with mixed-methods analysis. JMIR Ment Health.

[61] Klein B, Cook S (2010). Preferences for e-mental health services amongst an online Australian sample?. E J Applied Psychol.

[62] Casey L, Wright MA, Clough BA (2014). Comparison of perceived barriers and treatment preferences associated with internet-based and face-to-face psychological treatment of depression. Int J Cyber Behav Psychol Learn.

[63] Horgan A, Sweeney J (2010). Young students' use of the internet for mental health information and support. J Psychiatr Ment Health Nurs.

[64] Mohr DC, Siddique J, Ho J, Duffecy J, Jin L, Fokuo JK (2010). Interest in behavioral and psychological treatments delivered face-to-face, by telephone, and by internet. Ann Behav Med.

[65] Rizvi SL, Dimeff LA, Skutch J, Carroll D, Linehan MM (2011). A pilot study of the DBT coach: an interactive mobile phone application for individuals with borderline personality disorder and substance use disorder. Behav Ther.

[66] Watts S, Mackenzie A, Thomas C, Griskaitis A, Mewton L, Williams A, Andrews G (2013). CBT for depression: a pilot RCT comparing mobile phone vs. computer. BMC Psychiatry.

[67] Kenny R, Dooley B, Fitzgerald A (2015). Feasibility of "CopeSmart": a telemental health app for adolescents. JMIR Ment Health.

[68] Andersson G, Cuijpers P (2009). Internet-based and other computerized psychological treatments for adult depression: a meta-analysis. Cogn Behav Ther.

